# Symptomatic Tornwaldt Cyst: A Case Report

**DOI:** 10.7759/cureus.58796

**Published:** 2024-04-22

**Authors:** Stefan Konsulov, Taniel Minkov, Dimitar Pazardzhikliev, Denis Milkov, Daniel Markov

**Affiliations:** 1 Department of Otorhinolaryngology, Medical University of Plovdiv, Plovdiv, BGR; 2 Department of Otorhinolaryngology, University Hospital Kaspela, Plovdiv, BGR; 3 Department of General and Clinical pathology, Medical University of Plovdiv, Plovdiv, BGR

**Keywords:** endoscopic transnasal approach, embryonic notochord tumors, bilateral hearing loss, nasopharyngeal neoplasm, tornwaldt cyst

## Abstract

Thornwaldt cyst is a rare cystic formation, located along the midline of the nasopharynx. We present the case of a 60-year-old man with impaired nasal breathing and a several months-long history of serous otitis media. His only concomitant disease was arterial hypertension. The diagnostic imaging tests revealed a well-rounded cystic formation involving the upper part of the nasopharynx, characteristic of Thornwaldt cyst. Following, endoscopic transnasal marsupialization was performed and the benign cystic nature was confirmed on histopathology. The patient responded to the administered treatment and reported no persistence or emergence of new symptoms. The current case presents a symptomatic Thornwaldt cyst successfully treated by endoscopic transnasal marsupialization.

## Introduction

Thornwaldt cyst (also known as Tornwaldt, Thornwald, or nasopharyngeal cyst) is a rare cystic formation, most often located along the midline of the nasopharynx. These benign lesions are small in size and usually asymptomatic, only to be found incidentally on imaging studies or postmortem autopsies [1-3]. In some cases, Thornwaldt cysts can increase their size or become inflamed, leading to clinical symptoms, such as postnasal discharge, halitosis, Eustachian tube dysfunction, and headache [1]. The diagnosis is based on the medical history and diagnostic imaging (nasoendoscopy, computed tomography, or magnetic resonance imaging (MRI)) and confirmed by histopathology [1]. Thornwaldt cysts usually do not necessitate treatment, but in the emergence of clinical symptoms, they must be surgically excised [1,2]. We present the case of a symptomatic Thornwaldt cyst, treated successfully via endoscopic transnasal marsupialization.

## Case presentation

We present the clinical case of a 60-year-old man admitted to the outpatient Department of Otorhinolaryngology with complaints of bilateral hypacusis over the past months as well as predominantly left-sided impaired nasal breathing. The patient was initially treated with nasal decongestants and topical corticosteroid sprays, as well as mucolytics. Despite the applied treatment, the symptoms persisted, and additionally, periodic posterior rhinorrhea emerged. The nasal endoscopy revealed asymmetric hypertrophy of the epipharyngeal mucosa as well as a cystic formation in the region of the right lateral column, protruding toward the ipsilateral choana. The otoscopy revealed an adhesive left tympanic membrane, through which fluid was visible in the tympanic cavity. The patient did not suffer from any accompanying diseases, except for arterial hypertension, for which he is medicinally treated.

**Figure 1:**
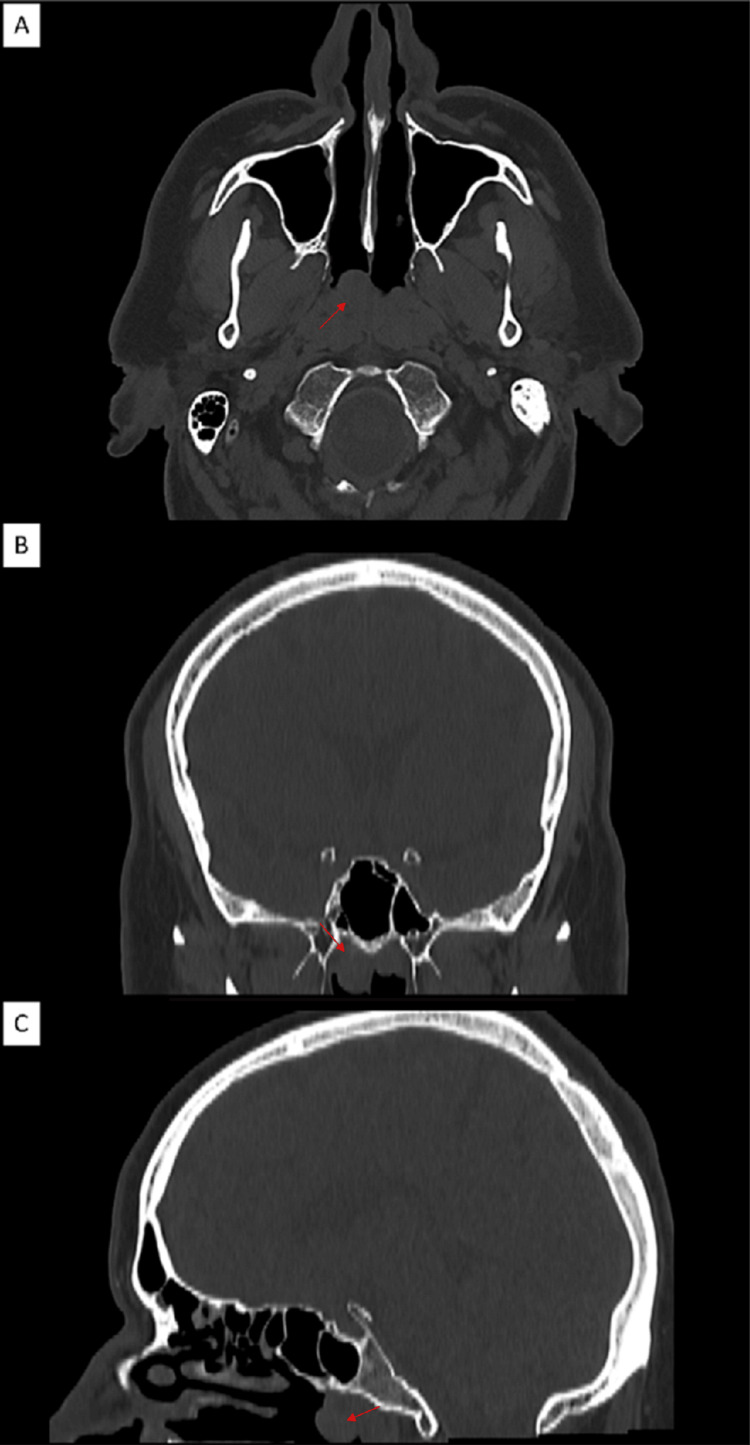
Computed tomography of a symptomatic Tornwaldt cyst. A. Axial view. B. Coronal view. C. Sagittal view. Red arrows show a well-rounded lesion in the nasopharynx.

The patient underwent a computed tomography scan of the head, which confirmed the hypertrophy of the posterior wall of the nasopharynx as well as the protrusion into the right choana of a well-rounded mass (Figure 1).

**Figure 2:**
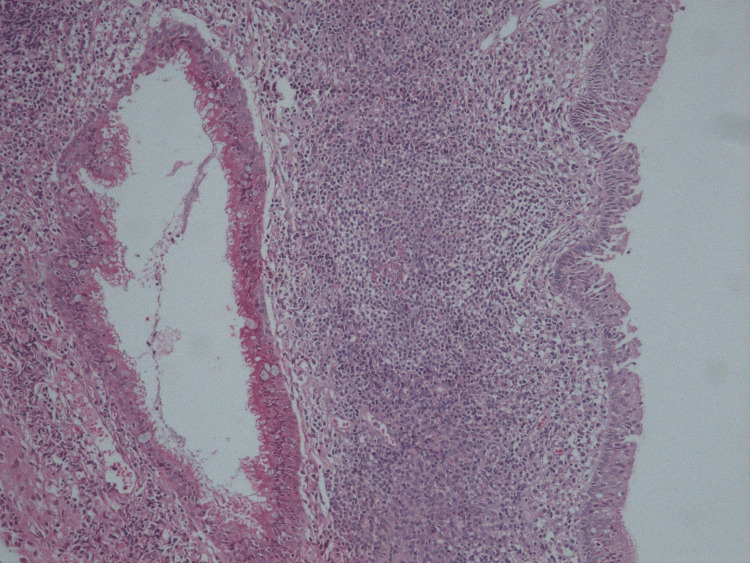
Pathohistology of a symptomatic Tornwaldt cyst. Wall of a benign cyst lined by respiratory-type epithelium with underlying lymphoid tissue and without any specific cells and layers (Hematoxylin-Eosin x200).

Due to the symptomatic clinical presentation, the patient underwent endoscopic marsupialization of the cystic formation involving the epipharynx, and a tympanostomy tube was placed in the left tympanic membrane. During the marsupialization, about 2-3 mL of exudate leaked out. The histological examination confirmed the benign nature of the formation in the nasopharynx (Figure 2).

In view of the history of the disease, the symptoms on admission, the results of the endoscopic examination, computed tomography, and pathological results, the diagnosis of Thornwaldt cyst was made.

**Figure 3:**
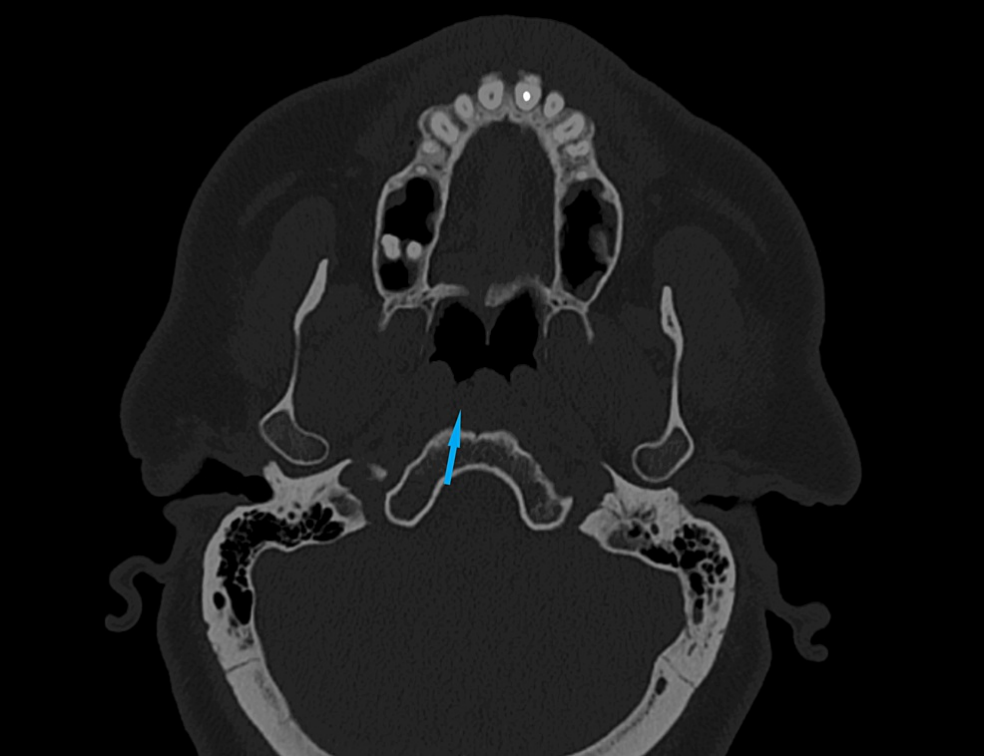
Computed tomography six months post-endoscopic marsupialization of Tornwaldt cyst. Blue arrow showing the absence of a lesion in the nasopharynx.

Six months postoperatively, the patient's symptoms had completely disappeared. A follow-up computed tomography showed no evidence of recurrence of the cystic formation (Figure 3). The case was studied in accordance with the Declaration of Helsinki.

## Discussion

Thornwaldt cyst is a dilatation of the pharyngeal bursa, an embryological remnant of the connection between the notochord and nasopharynx. In some cases, it may develop as a result of nasopharyngitis, chemoradiation, adenoidectomy, or other surgical trauma [1,2].

In 1890, Mayer first described a cystic mass on the posterior wall of the epipharynx [4]. The disease, however, is named after the German physician Gustav Ludwig Thornwald, who in 1885 described a series of 26 cases of nasopharyngeal cysts and the methods of their treatment [5]. Later, in 1912, Huber described the relation of the notochord to the anlage of the pharyngeal bursa or the median pharyngeal recess, anatomically naming it a “potential space” [6].

Thornwaldt cysts are usually asymptomatic and often diagnosed incidentally on imaging studies and autopsies, with an incidence rate of 0.2-5% and 4%, respectively [1,7]. They are usually discovered between the ages of 15 and 60, with no particular sex predilection [1]. In some cases, however, they can become infected and/or enlarged, leading to symptoms such as epipharyngeal discharge, occipital headache, halitosis, Eustachian tube dysfunction with middle ear effusion, and hearing loss [7,8]. The patient we present is 60 years old and had such symptoms.

The diagnosis is made by endoscopy and other imaging studies, such as computed tomography and MRI [1-3,7,8]. Therefore, it is imperative to know the landmarks in this region, which may be obliterated by disease, and radiologists should be aware of this entity or remnant [9]. On non-contrast-enhanced computed tomography, a low-density cyst on the posterior wall of the nasopharynx is suggestive of a Thornwaldt cyst. In contrast-enhanced computed tomography, while the rim of the cyst may be enhanced, the cyst itself shows low attenuation. MRI remains the diagnostic method of choice for the detection of these lesions due to its superiority over computed tomography in terms of delineating soft tissues. T1-weighted MRI scans show intermediate to high signal intensity and T2-weighted images show high signal intensity of a well-rounded mass along the midline of the posterior pharyngeal wall [7,8,10-12]. In the case we described, the protrusion predominated on one side.

Pathologically, the walls of the Thornwaldt cyst are covered with respiratory epithelium, are poorly infiltrated by lymphocytes, and lymph follicles are absent. The cyst contents are usually high in protein [13,14]. Unfortunately, the protein content of the cyst was not evaluated in our case.

The differential diagnosis of Thornwaldt cyst includes adenoid hyperplasia, adenoid rest, intra-adenoid cyst, retention cyst, branchial cleft cyst, Rathke's cleft cysts, meningocele, sphenoid sinus mucocele, retropharyngeal abscess, and nasopharyngeal carcinoma [1,2,7,8]. Branchial cleft cysts are usually located in the lateral aspect of nasopharyngeal space, but Thornwaldt cysts, adenoid cysts, and Rathke's cleft cysts are found near the midline [8]. Rathke’s cleft cyst has an internal stratified squamous lined epithelium and is typically found in the sellar or suprasellar region [1,2]. The differential diagnosis in our case was based in regard to the location, diagnostic imaging appearance, and histopathology.

Asymptomatic cysts need not be surgically treated but only followed regularly. When the cyst is large and causes complaints, surgical resection and marsupialization and laser surgery are possible methods of treatment [15]. Both transoral and transnasal endoscopic approaches can be performed with the latter used in our case. The endoscopic technique provides a superior view of the surgical field, allowing meticulous marsupialization of the Thornwaldt cyst, while avoiding damage to the orifices of the Eustachian tube, and allows for a low risk of recurrence [1,8,10].

## Conclusions

The diagnosis of Thornwaldt cyst is based on diagnostic imaging and histopathological examination. Although it may be asymptomatic and not require treatment, the Thornwaldt cyst in our case was associated with unilateral nasal obstruction, posterior rhinorrhea, and Eustachian tube dysfunction. We successfully treated the patient by endoscopic transnasal marsupialization with symptom resolution and no recurrence six months postoperatively.

## References

[1] Canatan MO, Canatan MF, Canatan AN (2023). An incidental Tornwaldt cyst finding on the postoperative assessment of a nasal septum deviation: a case report. Cureus.

[2] Yuca K, Varsak YK (2012). Thornwaldt’s cyst. Eur J Gen Med.

[3] Moody MW, Chi DH, Mason JC, Phillips CD, Gross CW, Schlosser RJ (2007). Tornwaldt's cyst: incidence and a case report. Ear Nose Throat J.

[4] Mayer FJC (1842). Neue Untersuchungen aus dem Gebiete der Anatomie und Physiologie.

[5] Tornwaldt GL, Bergmann JF, Krankheiten W (1885). Uberdi e Bedeutung der bursa pharygea furdieerkennung und behandlung gewisse r nasen rauchenraum.

[6] Huber CC (1934). On the relation of the chorda dorsalis to the anlage of the pharyngeal bursa or the median pharyngeal recess. Anat Rec.

[7] Baisakhiya N, Deshmukh P, Pawar V (2011). Tornwaldt cyst: a cause of neck pain and stiffness. Indian J Otolaryngol Head Neck Surg.

[8] Lee JH (2021). Huge Tornwaldt cyst with otitis media with effusion. Ear Nose Throat J.

[9] Sindel A, Turhan M, Ogut E, Akdag M, Bostancı A, Sindel M (2014). An endoscopic cadaveric study: accessory maxillary ostia. Dicle Medical Journal/Dicle Tıp Dergisi.

[10] Turan Ş, Gürbüz MK, Kaya E, Pinarbaşli MÖ, Uzun T, Çakli H (2020). Is transnasal endoscopic marsupialization sufficient in Thornwaldt cysts?. J Craniofac Surg.

[11] Toufga Z, Fikri M (2019). The Tornwaldt cyst. PAMJ - Clinical Medicine.

[12] Weissman JL (1992). Thornwaldt cysts. Am J Otolaryngol.

[13] Miyahara H, Matsunaga T (1994). Tornwaldt's disease. Acta Otolaryngol Suppl.

[14] Surjith V, Hudgins PA (2022). Tornwaldt cyst. Diagnostic Imaging Head and Neck.

[15] Caliman MA, Cabernite EM, Vieira JT, Pasin DC, Fomin DS (2013). Thornwaldt cyst - treatment with diode laser. Braz J Otorhinolaryngol.

